# Selection of standards for estimated fetal weight percentiles

**DOI:** 10.1002/pmf2.70337

**Published:** 2026-06-04

**Authors:** C. Andrew Combs, Olaide Ashimi Balogun, Sushma Amara, Zachary S. Bowman, Peter A. Combs

**Affiliations:** ^1^ Pediatrix Center for Research, Education, Quality and Safety, Pediatrix & Obstetrix Sunrise Florida USA; ^2^ Obstetrix Maternal‐Fetal Medicine Specialists of Houston, part of Pediatrix & Obstetrix Houston Texas USA; ^3^ Eastside Maternal‐Fetal Medicine Specialists, part of Pediatrix & Obstetrix Bellevue Washington USA; ^4^ Perinatal Associates of Sacramento, part of Pediatrix & Obstetrix Sacramento California USA; ^5^ Independent Statistical Consultant San Francisco California USA; ^6^ Present address: Gould Medical Group Stockton California USA

**Keywords:** birth weight references, fetal biometry, fetal growth restriction, fetal weight standards, large for gestational age, small for gestational age

## Abstract

**Objectives:**

To evaluate which of 10 standards or references should be used for determination of percentiles of estimated fetal weight (EFW).

**Study design:**

Cross‐sectional study using pooled data from four maternal‐fetal medicine (MFM) practices. Inclusion criteria were as follows: ultrasound exam during 2022, singleton fetus, gestational age 24^0/7^ to 39^6/7^ weeks, biometry measured, and fetal cardiac activity present. EFW was calculated using the three‐parameter formula of Hadlock et al. We evaluated five American birth weight (BW) references and five fetal weight (FW) standards. The fit of each reference or standard to our EFW distribution was assessed by seven criteria: mean *z*‐score close to 0, standard deviation (SD) of *z* close to 1, Kolmogorov–Smirnov D‐statistic close to 0, close to 10% of EFWs <10th percentile, close to 10% of EFWs > 90th percentile, and Youden J‐statistics close to 1 at the 10th and 90th percentiles.

**Results:**

In 20,643 ultrasound exams, the formulas from the 1991 FW standard of Hadlock et al. had the best fit to our distribution of EFW (mean *z*‐score 0.07, SD of *z* 1.04, D‐statistic 0.036, J‐statistics 0.946 and 0.972 at 10th and 90th percentile, respectively). All five EFW standards had better fit than any of the BW references, primarily because the range of normal of the BW references was much larger than the normal range of observed EFWs, resulting in underdiagnosis of abnormal EFW (both EFW <10th percentile and EFW >90th percentile). The range of normal from the Hadlock table was larger than that from the Hadlock formulas and the observed range, resulting in fewer diagnoses of abnormal EFW. In the subgroup of patients at low risk for small for gestational age or large for gestational age, the Hadlock formulas still had the best fit to our EFW distribution.

**Conclusions:**

Of the norms studied, the FW distribution from the Hadlock 1991 formulas had the best fit to our observed EFW distribution and are the most appropriate standard for determination of EFW percentiles in our US‐based MFM practices. All the FW standards had better fit to our data than any of the BW references, so EFW percentiles should be based on FW standards, especially the Hadlock standard, not BW references.

## 1 INTRODUCTION

Evaluation of estimated fetal weight (EFW) is an essential component of second‐ and third‐trimester obstetrical ultrasound examinations [1]. Two distinct steps are required to determine whether EFW is normal or abnormal. First, EFW is calculated from biometric measurements such as biparietal diameter (BPD), head circumference (HC), abdominal circumference (AC), and femur length (FL). Second, an EFW percentile is determined from norms that specify the distribution of fetal weight (FW) at each gestational age (GA). The normal range is typically defined as the 10th to the 90th percentile.

There are at least 70 published formulas for calculating EFW from fetal biometry [2]. Of these, the three‐parameter (HC, AC, FL) formula of Hadlock et al. [3] has been consistently shown to produce the most accurate prediction of birth weight (BW) [2, 4]. We recently reported that this formula yields accurate predictions of BW even when ultrasound is performed more than 2 months before birth [5].

For determination of EFW percentile, there are at least 2 dozen norms that can be used [6, 7, 8, 9, 10, 11, 12, 13, 14, 15, 16, 17, 18, 19, 20, 21, 22, 23, 24, 25, 26, 27, 28, 29, 30, 31, 32]. Some of these are based on BW and some are based on FW. Some norms provide tables showing selected GA‐specific percentiles (10th, 50th, 90th, and often other percentiles). Other norms provide formulas of mean and standard deviation (SD), from which one can calculate percentiles. Tables of BW distributions are most often “references” which means they are derived from whole populations, typically from a single country or region. Tables or formulas of FW distributions are often “standards” which means they are derived from healthy subsets of the population without risk factors for altered fetal growth such as hypertension, diabetes, or other health issues [33]. A few standards are race‐ and ethnicity‐specific [27].

Whether a given value of EFW is considered normal or abnormal depends on which reference or standard is used. Ultrasound machines and reporting software allow users to choose between several norms, but there is little existing guidance regarding how to select which norm to use. For diagnosis of fetal growth restriction (FGR), the Society for Maternal‐Fetal Medicine (SMFM) recommends using a norm based on FW rather than one based on BW [34]. In part, this is because BW may be biased toward lower values at early GAs due to a variety of conditions associated with small‐for‐GA (SGA) BW that are also associated with early preterm birth (e.g., FGR or hypertensive disorders).

Ideally, the norm used by an ultrasound practice will have a mean value and range of normal close to the mean and normal range of the population examined in the practice. Salomon et al. suggested a method using *z*‐scores and related statistics to evaluate how well a practice's biometric distribution fits with a given norm [35]. Sananes et al. demonstrated how the method might be applied to evaluate various norms for BPD, HC, FL, and AC in their practice [36]. Both groups concluded that evaluating the concordance between a study population and selected norms should be the first step in quality control. In other words, each institution should use the Salomon method or other, similar methods to evaluate the norms they use.

The aim of the present study was to evaluate which of 10 references or standards should be used for determination of EFW percentiles in our maternal‐fetal medicine (MFM) practices. We adapted the methods of Salomon et al. [35] to compare our observed distribution of EFW to the distribution predicted by each reference or standard.

## 2 MATERIALS AND METHODS

In this cross‐sectional study, we pooled ultrasound exam findings from four MFM practices in the southern and western United States. The practices were accredited for obstetrical ultrasound by the American Institute of Ultrasound in Medicine. Sonographers were certified by the Registry of Diagnostic Medical Sonographers. Sonologists were board‐certified or board‐eligible MFM subspecialists. We used Voluson (GE Healthcare) or Philips Affiniti ultrasound machines (Philips Electronics). Exam reports were generated using GE Viewpoint™ software, findings were stored in a secure online computer database, and exam data were extracted for routine quality audit. Analysis and reporting of the deidentified retrospective data were determined to be exempt by the Institutional Review Board of the Good Samaritan Hospital, San Jose, California.

Inclusion criteria for this study were as follows: ultrasound exam from January to December 2022, singleton pregnancy, fetal cardiac activity present, four standard fetal biometry measurements recorded (BPD, HC, AC, and FL), and GA 24^0/7^ to 39^6/7^ weeks. Exact GA (weeks + days/7) was calculated based on best obstetrical estimate, typically following guidelines of the American College of Obstetricians and Gynecologists [37]. The 24‐week lower GA limit was selected because some BW references excluded GA <24 weeks and the <40‐week upper GA limit was selected because we had a paucity of exams at GA ≥40 weeks. The present data are a subset of exams reported in prior investigations of fetal biometry [38, 39] which included additional exams from <24 weeks GA.

For this study, EFW was calculated using the 1985 three‐parameter formula of Hadlock et al. (using HC, AC, and FL) regardless of which formula was used on the ultrasound report [3]. This formula was chosen because prior investigations concluded that it is the most accurate formula of several studied [2, 4] and yields reliable predictions of BW up to 3 months before birth [5].

We evaluated the fit between our observed EFW distribution and the distribution predicted by five US BW references (Brenner et al. [6], Williams et al. [7], Alexander et al. [13], Olsen et al. [24], and Duryea et al. [26]) and five FW standards (Hadlock et al. from 1991 formulas and table [10], the World Health Organization [WHO] Fetal Growth Charts [29], the INTERGROWTH‐21st Project [IG‐21st] [30], and the National Institutes of Child Health and Human Development Fetal Growth Studies Unified Standard [NICHD‐U] [31, 32]). For the Hadlock [10], IG‐21st [30], and NICHD‐U [32] standards, we used the published formulas for mean and variance to calculate *z*‐scores and the 10th and 90th percentiles. For all the other norms, we used the 10th, 50th, and 90th percentiles from the published data tables; we also included an evaluation of the data table published with the Hadlock standard [10] because recent publications have reported that the formulas and the table have different cut‐off values for the 10th and 90th percentiles [32, 40]. For all norms, we defined the range of normal as the 90th percentile minus the 10th percentile.

To provide a visual comparison to our EFW distribution, we plotted the mean weight and the range of normal for the 10 norms and observed EFW as a function of GA.

A *z*‐score for each exam was calculated for each norm as $z=\left(\mathrm{EFW}-{\mathrm{mean}}_{\mathrm{norm}}\right)/{\mathrm{SD}}_{\mathrm{norm}}$, where mean_norm_ and SD_norm_ are the GA‐specific 50th percentile and standard deviation, respectively. For norms based on table data, we took SD as the range of normal divided by 2.5632 because the 10th percentile of a normal distribution is 1.2816 SD below the mean and the 90th percentile is 1.2816 SD above the mean, a total of 2.5632 SD for the entire range. For norms based on table data, linear interpolation was used for non‐integer values of GA.

For each norm, we calculated the mean and SD of *z*‐score and we plotted histograms comparing the observed and expected (ideal) distribution of *z*‐score. To test goodness of fit, we evaluated how close the mean and SD *z*‐score were to the expected ideal values of 0 and 1, respectively. We also calculated the Kolmogorov–Smirnov D‐statistic as suggested by Salomon et al. [35]; values of D close to 0 indicate a good fit to a standard normal distribution. Additional assessments of goodness of fit included evaluation of the percentage of exams in the tails of the distributions (<10th and >90th percentiles, with expected values of 10% in each tail) and the Youden J‐statistic at each of these tail cut‐offs as suggested by Salomon et al. [35]; values of J close to 1 indicate a good fit. These evaluations were based on observations from all GAs combined.

To understand the effect of GA on the observations, we also plotted the percentage of exams where EFW was abnormal (i.e., <10th percentile, >90th percentile) as a function of GA.

To approximate a standard population, we condensed the hundreds of specific indications for the exams into 22 categories as previously described [38, 39, 41] and categorized each exam into one of four mutually exclusive risk groups: risk factors for large for GA (LGA), risk factors for SGA, risk factors for both LGA and SGA, or risk factors for neither (“low‐risk” subgroup). Using the low‐risk subgroup as an approximation to a standard population, we repeated the analyses of mean and SD of *z*‐score, D‐statistic, J‐statistics, and percentage of abnormal exams in each tail.

Analyses were performed using Stata version 18.0 software (StataCorp). For patients with more than one eligible exam during the year, we randomly selected one exam per patient by using the Stata function “runiform()” to generate a uniformly distributed pseudorandom number between 0 and 1 for each exam; we multiplied that number times the number of replicate exams per patient, truncated the result to an integer (*n*), added 1, and selected the (*n*+1)th replicate for inclusion. Published formulas for the NICHD‐U standard [32] were coded in R script, so R software (R Foundation for Statistical Computing) was used to calculate the GA‐specific natural logarithm of mean_norm_ and SD_norm_ which were then merged into the Stata data file.

Mean *z*‐score was compared to the ideal of 0 using one‐sample *t*‐test. Percentage of exams in the tails of the distributions (<10th percentile, >90th percentile) were compared to ideal expected values (10% for both) using one‐sample *z*‐test. Percentage of exams in the tails were also compared between the 10 norms using two‐sample *z*‐test. Nominal significance level was set at two‐tailed *p*‐value <0.05. Because of multiple comparisons, Bonferroni adjustment to *p* < 0.005 was made for the 10 one‐sample *t*‐tests and the 10 one‐sample *z*‐tests and adjustment to *p* < 0.0011 was made for the 45 pairwise two‐sample *z*‐tests.

## 3 RESULTS

**FIGURE 1:**
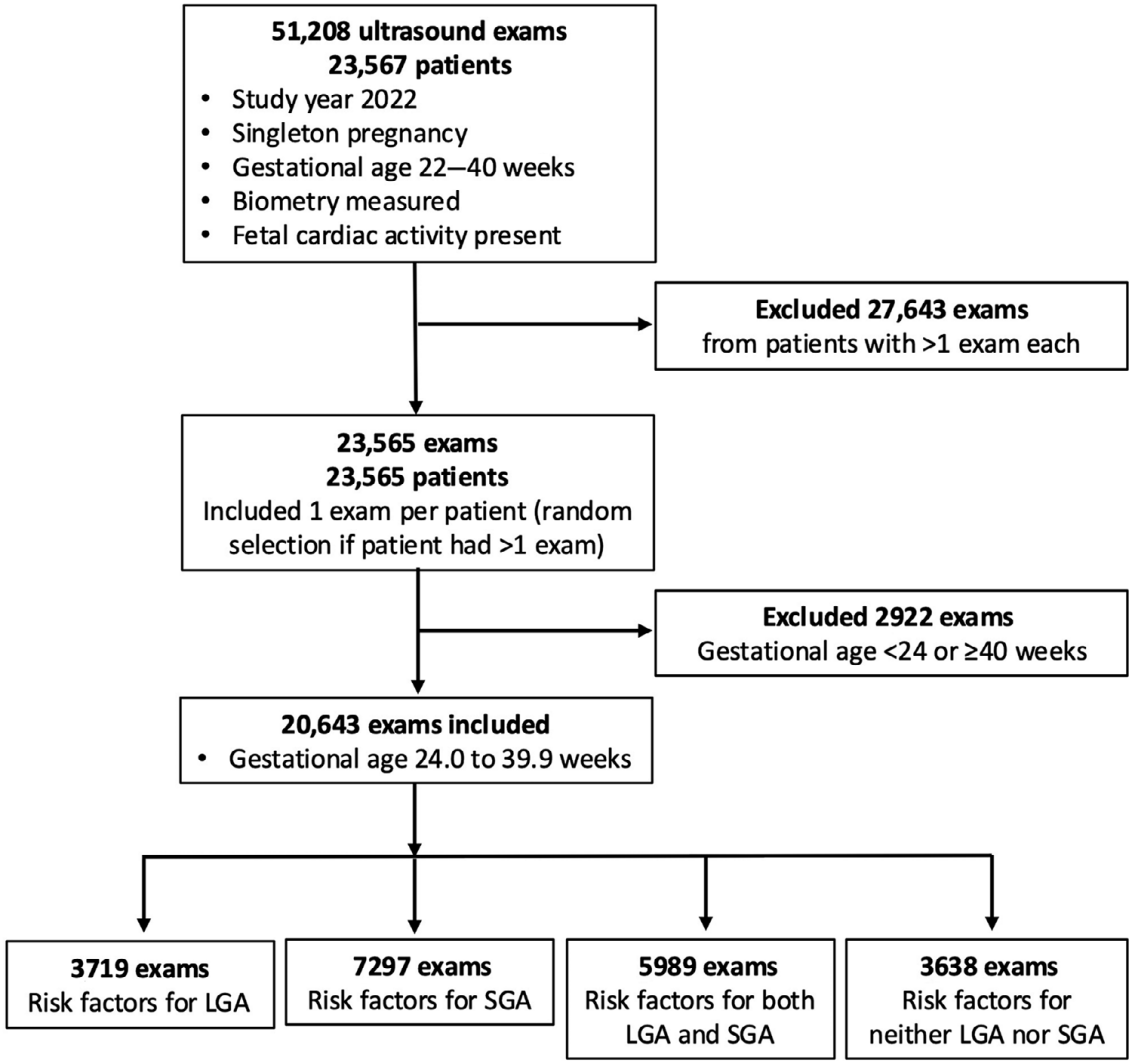
Flow diagram showing number of ultrasound exams meeting inclusion and exclusion criteria. LGA, large for gestation age; SGA, small for gestation age.

During 2022, 20,643 patients had one or more ultrasound exams meeting inclusion criteria. After random selection of one exam per patient for patients with multiple exams, 20,643 exams were included in the analysis. Figure 1 is a flow diagram showing the number of patients meeting inclusion or exclusion criteria. Table 1 summarizes characteristics of the included population.

**TABLE 1:** Characteristics of study population at the time of the ultrasound exam.

Characteristic	Value
Gestational age, weeks, median (IQR)	32.2 (28.6‐34.9)
Gestational age group	
24–27.9 weeks	4062 (19.7%)
28–31.9 weeks	4892 (23.7%)
32–35.9 weeks	8163 (39.5%)
36–40 weeks	3529 (17.1%)
Maternal age, years, mean ± standard deviation	30.2 ± 6.1
Maternal age group	
<25 years	3,0356 (17.3%)
25–29.9 years	4927 (23.9%)
30–34.9 years	5958 (28.9%)
35–39.9 years	4793 (23.2%)
≥40 years	1389 (6.7%)
Maternal body mass index	
<30 kg/m^2^	13,128 (63.6%)
30–39.9 kg/m^2^	5326 (25.8%)
≥40 kg/m^2^	2189 (10.6%)
Maternal self‐reported race	
Asian	2038 (9.9%)
Black	2748 (13.3%)
Hispanic	3918 (19.0%)
White	5498 (26.6%)
Multiple or Other	249 (1.2%)
Not reported	6195 (30.0%)
Health insurance	
Commercial	8009 (38.8%)
Medicaid or other government	10,648 (51.6%)
Other	77 (0.4%)
Not recorded	1909 (9.2%)
Diabetes mellitus	
None	17,482 (84.7%)
Gestational	2432 (11.8%)
Type 2	635 (3.1%)
Type 1	94 (0.5%)
Hypertensive disorders	
None	18,953 (91.8%)
Chronic hypertension without preeclampsia	1337 (6.5%)
Gestational hypertension	239 (1.2%)
Preeclampsia or superimposed preeclampsia	114 (0.6%)
Recorded substance use	
Tobacco	38 (0.2%)
Alcohol	11 (0.1%)
Other	42 (0.2%)
Prior pregnancy history	
Fetal growth restriction	79 (0.4%)
Preeclampsia	247 (1.2%)
Preterm birth	278 (1.4%)
Fetal congenital anomaly	285 (1.4%)
Repetitive miscarriages	23 (0.1%)
Risk group	
Risk factors for LGA	3719 (18.0%)
Risk factors for SGA	7297 (35.4%)
Risk factors for both LGA and SGA	5989 (29.0%)
Risk factors for neither LGA nor SGA	3638 (18.0%)

Abbreviations: IQR, interquartile range; LGA, large for gestational age; SGA, small for gestational age.

**FIGURE 2:**
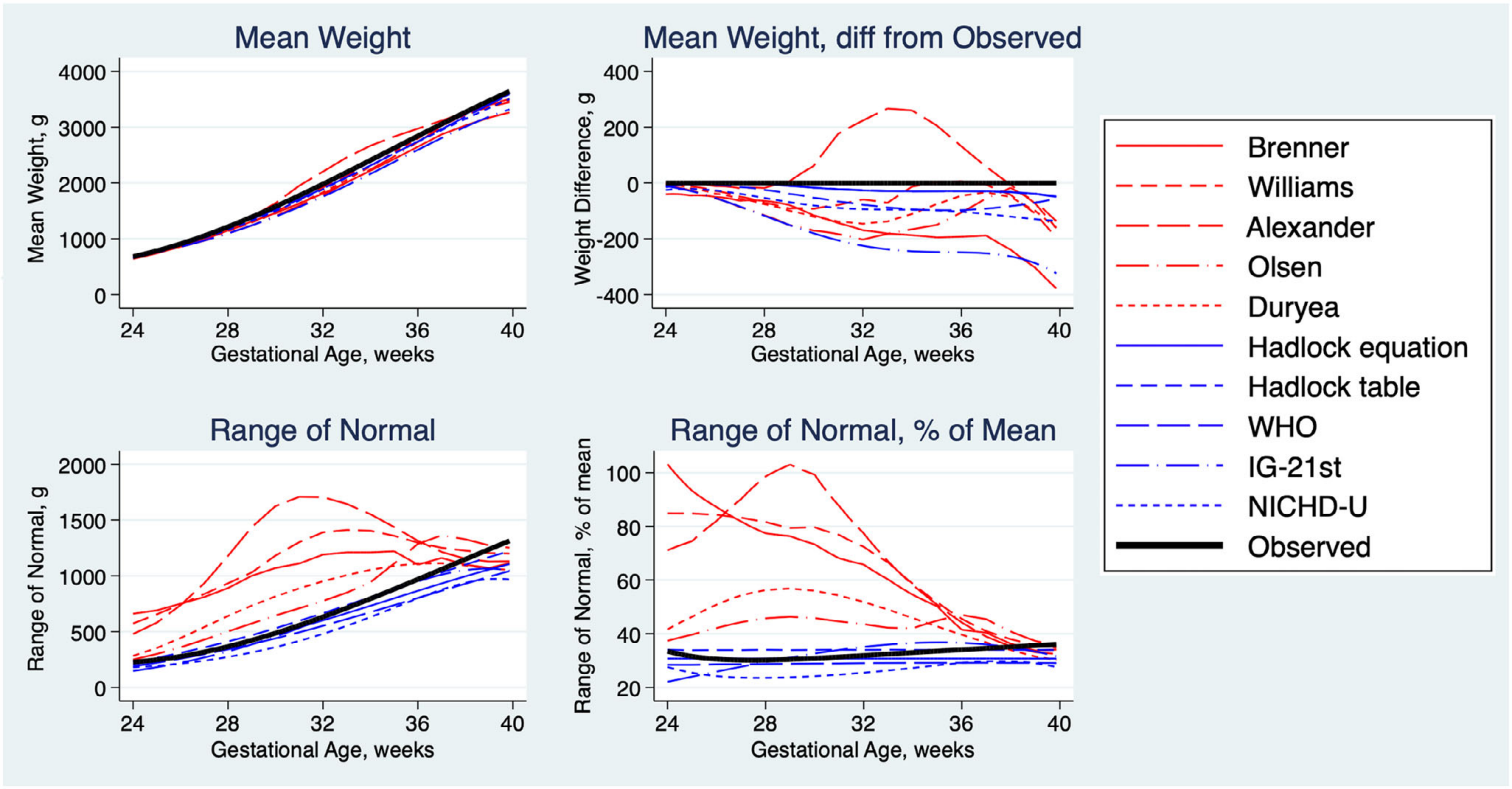
Mean and range of normal for 10 established fetal weight norms and our observed estimated fetal weights (using the three‐parameter formula of Hadlock et al. 1985 [3]). Range of normal is 90th percentile minus 10th percentile. Red curves are derived from birth weight references, blue curves from fetal weight standards, and black curves from our data (*N* = 20,643). IG‐21st, INTERGROWTH‐21st Project; NICHD‐U, National Institutes of Child Health and Human Development Fetal Growth Studies Unified Standard; WHO, World Health Organization.

Figure 2 shows the mean and range of normal for each of the 10 norms and our observations. Although the mean values appear roughly similar at the scale of the upper left panel, the upper right panel reveals differences in the means which increase with GA. Our observed mean EFW appears to be larger than all the norms except the Alexander reference [13]. For the means, the BW references (red curves) are generally similar to the FW standards (blue curves). However, all the BW references had a much larger range of normal than the FW standards, as shown in the lower panels. For the all the FW standards (blue), the range of normal was about 30% of mean weight and fairly constant across GA (lower right panel). In contrast, the range of normal for the BW references varied from 40‐100% of mean weight and generally decreased across GA.

**FIGURE 3:**
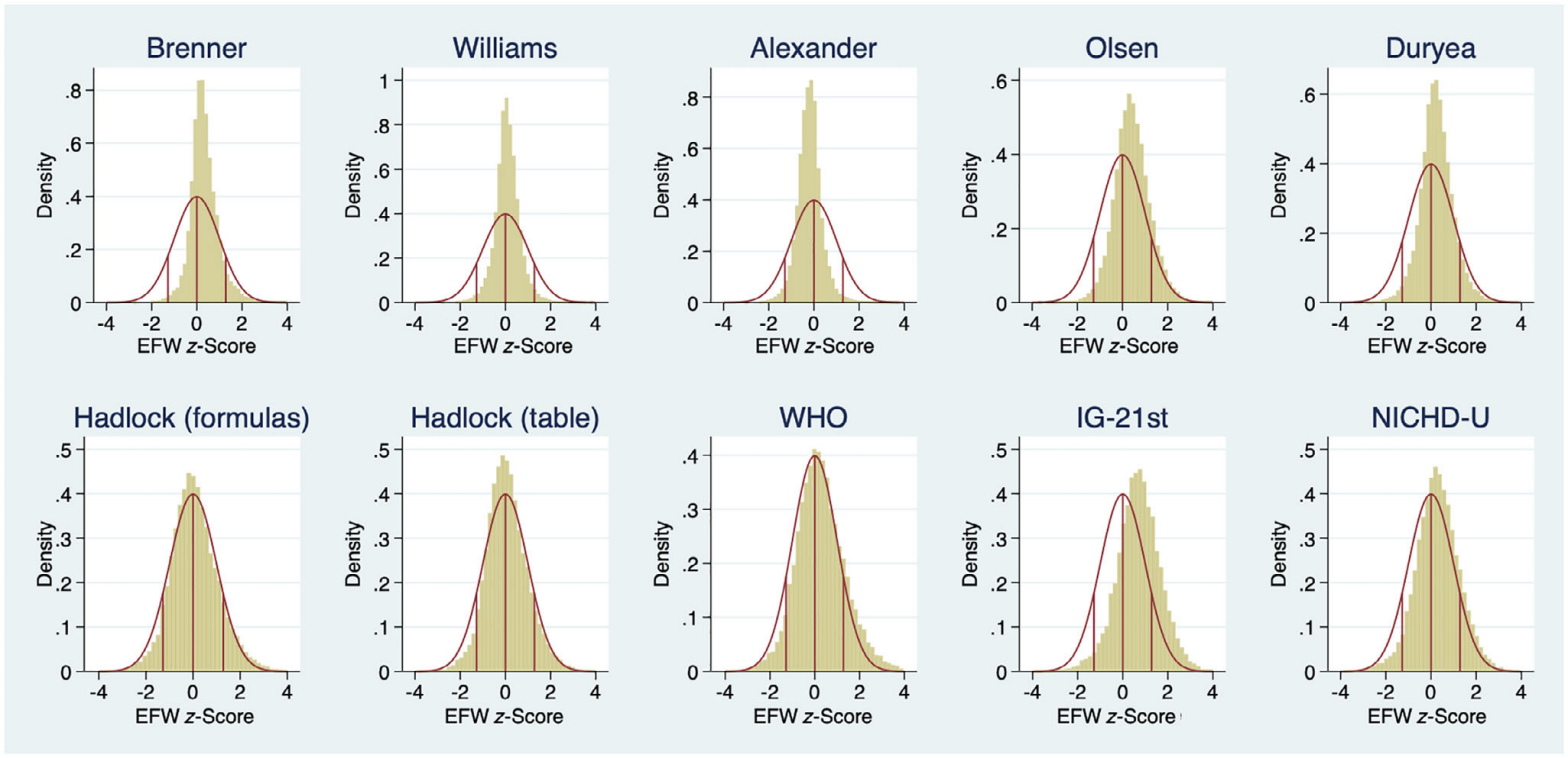
Histograms of observed estimated fetal weight (EFW) *z*‐score (bars) compared with 10 established weight norms (red curves). Drop lines plotted at 10th, 50th, and 90th percentiles (left to right). Upper row is based on birth weight references, lower row is based on fetal weight standards. IG‐21st, INTERGROWTH‐21st Project; NICHD‐U, National Institutes of Child Health and Human Development Fetal Growth Studies Unified Standard; WHO, World Health Organization.

The overall fit of EFW *z*‐scores to the 10 norms is summarized in Table 2 and illustrated by the histograms in Figure 3. A perfect fit of observations to a given norm would result in a mean *z*‐score of 0 and SD of 1. The Hadlock standard [10] came closest to these ideals whether based on the published formulas or the table. The other three FW standards had a larger mean value of *z*‐score, manifest as a shift to the right of the histograms, meaning that our observed values averaged higher than the standards. For all the BW references, the SD of *z*‐score was far less than 1, meaning that the variance of the reference was much larger than the variance of the observations, consistent with the lower panels of Figure 1 and evident by inspection of the histograms in Figure 3, where the red BW reference curves have a much wider distribution than the observed values. As a result, the percentage of observations in the tails of the distribution for the BW references was much less than the ideal 10%. For all the FW standards, the SD of *z*‐score was very close to 1, so the percentage of observations in the tails was closer to 10% than for the BW references. The Hadlock formulas and table [10] had the lowest values of D, indicating a good fit to a standard normal distribution. The Hadlock formulas and table [10] also had values of J‐statistic closest to 1, reflecting the best overall performance of combined sensitivity and specificity.

**TABLE 2:** Comparison of 10 norms for estimated fetal weight in all exams (*N* = 20,643).

Reference or standard	Year	Source	Type	Mean *z*‐score	Standard deviation of *z*‐score	Kolmogorov–Smirnov D‐statistic	Exams with EFW <10th percentile	Youden J‐statistic at 10th percentile	Exams with EFW >90th percentile	Youden J‐statistic at 90th percentile
Ideal values				0	1	0	2064 (10%)	1	2064 (10.0%)	1
Brenner [6]	1976	Table	BWR	0.33^a^	0.63	0.275	225 (1.1%)^B^, ^d^, ^e^, ^f^, ^g^, ^h^, ^I^, ^J^	0.152	1093 (5.3%)^B^, ^d^, ^e^, ^f^, ^g^, ^h^, ^I^, ^J^	0.449
Williams [7]	1982	Table	BWR	0.09^a^	0.56	0.199	324 (1.6%)^B^, ^c^, ^e^, ^g^, ^h^, ^I^, ^J^, ^K^, ^l^	0.219	437 (2.1%)^B^, ^c^, ^e^, ^f^, ^g^, ^h^, ^I^, ^K^, ^l^	0.224
Alexander [13]	1996	Table	BWR	−0.19^a^	0.56	0.239	709 (3.4%)^B^, ^c^, ^d^, ^f^, ^g^, ^h^, ^I^, ^J^, ^l^	0.477	253 (1.2%)^B^, ^c^, ^e^, ^f^, ^g^, ^h^, ^I^, ^K^, ^l^	0.127
Olsen [24]	2010	Table	BWR	0.43^a^	0.81	0.225	326 (1.6%)^B^, ^d^, ^e^, ^g^, ^h^, ^I^, ^J^, ^K^, ^l^	0.221	2853 (13.8%)^B^, ^c^, ^d^, ^e^, ^g^, ^h^, ^I^, ^J^, ^K^, ^l^	0.889
Duryea [26]	2014	Table	BWR	0.25^a^	0.74	0.187	488 (2.4%)^B^, ^c^, ^d^, ^e^, ^f^, ^h^, ^I^, ^J^, ^K^, ^l^	0.327	1432 (6.9%)^B^, ^c^, ^d^, ^e^, ^f^, ^h^, ^I^, ^J^, ^K^, ^l^	0.725
Hadlock [10]	1991	Formulas	FWS	0.08^a^	1.04	0.036	1485 (7.2%)^B^, ^c^, ^d^, ^e^, ^f^, ^g^, ^I^, ^K^, ^l^	0.940	2393 (11.6%)^B^, ^c^, ^d^, ^e^, ^f^, ^g^, ^I^, ^J^, ^K^, ^l^	0.970
Hadlock [10]	1991	Table	FWS	0.07^a^	0.94	0.059	1138 (5.5%)^B^, ^c^, ^d^, ^e^, ^f^, ^g^, ^h^, ^J^, ^K^	0.764	1969 (9.5%)^c^, ^d^, ^e^, ^f^, ^g^, ^h^, ^J^, ^K^, ^l^	0.948
WHO [29]	2017	Table	FWS	0.28^a^	1.13	0.084	1383 (6.7%)^B^, ^c^, ^d^, ^e^, ^f^, ^g^, ^I^, ^K^, ^l^	0.880	3330 (16.1%)^B^, ^c^, ^d^, ^e^, ^f^, ^g^, ^h^, ^I^, ^K^, ^l^	0.925
INTERGROWTH‐21st [30]	2017	Formulas	FWS	0.68^a^	1.03	0.294	628 (3.0%)^B^, ^c^, ^d^, ^f^, ^g^, ^h^, ^I^, ^J^, ^l^	0.370	5359 (26.0%)^B^, ^c^, ^d^, ^e^, ^f^, ^g^, ^h^, ^I^, ^J^, ^l^	0.817
NICHD Uniform [32]	2025	Formulas	FWS	0.28^a^	1.00	0.126	1063 (5.4%)^B^, ^c^, ^d^, ^e^, ^f^, ^g^, ^h^, ^J^, ^K^	0.719	2959 (14.3%)^B^, ^c^, ^d^, ^e^, ^f^, ^g^, ^h^, ^I^, ^J^, ^K^	0.945

Abbreviations: BWR, birth weight reference; EFW, estimated fetal weight; FWS, fetal weight standard; NICHD, National Institutes of Child Health and Human Development; WHO, World Health Organization.

^a^ Significantly different than 0, *p* < 0.005, *t*‐test.

^B^ Significantly different than 10%, *p* < 0.005, one‐sample *z*‐test.

^c^ Significantly different than Brenner, *p* < 0.0011, two‐sample *z*‐test.

^d^ Significantly different than Willaims, *p* < 0.0011, two‐sample *z*‐test.

^e^ Significantly different than Alexander, *p* < 0.0011, two‐sample *z*‐test.

^f^ Significantly different than Olsen, *p* < 0.0011, two‐sample *z*‐test.

^g^ Significantly different than Duryea, *p* < 0.0011, two‐sample *z*‐test.

^h^ Significantly different than Hadlock formulas, *p* < 0.0011, two‐sample *z*‐test.

^I^ Significantly different than Hadlock table, *p* < 0.0011, two‐sample *z*‐test.

^J^ Significantly different than WHO, *p* < 0.0011, two‐sample *z*‐test.

^K^ Significantly different than INTERGROWTH‐21st, *p* < 0.0011, two‐sample *z*‐test.

^l^ Significantly different than NICHD Uniform, *p* < 0.0011, two‐sample *z*‐test.

**FIGURE 4:**
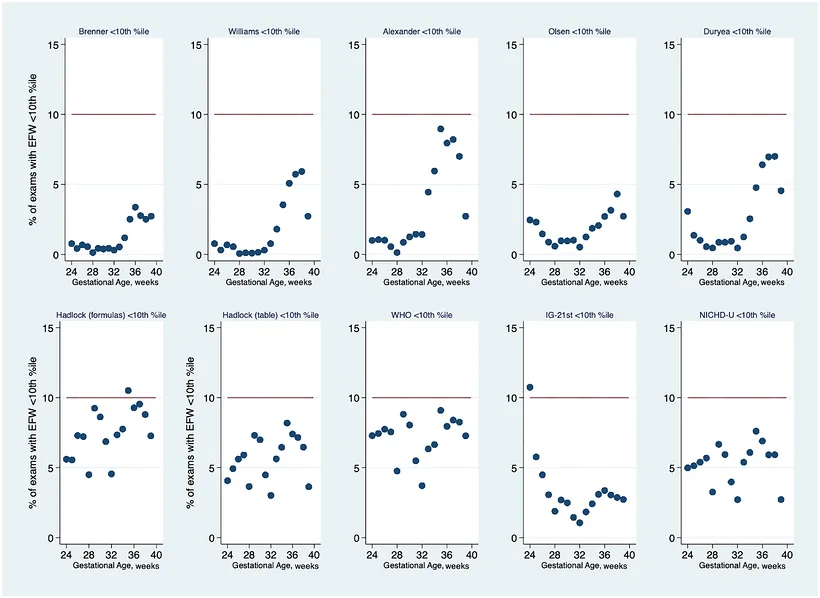
Percentage of exams with estimated fetal weight (EFW) less than the 10th percentile. Observed frequency at each completed week (blue dots). Red lines plotted at 10%, the “ideal” expected frequency. IG‐21st, INTERGROWTH‐21st Project; NICHD‐U, National Institutes of Child Health and Human Development Fetal Growth Studies Unified Standard; WHO, World Health Organization.

**FIGURE 5:**
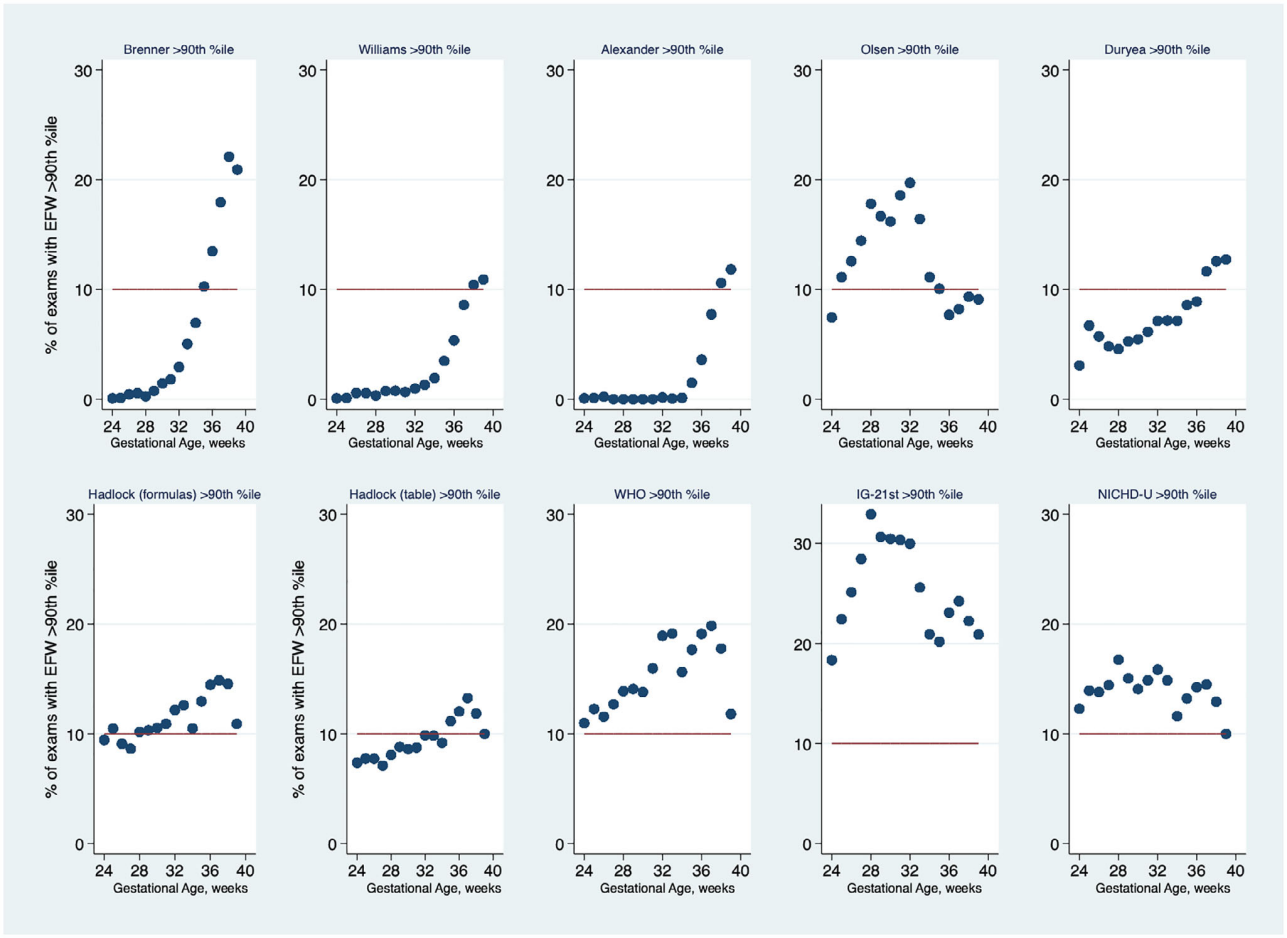
Percentage of exams with estimated fetal weight (EFW) greater than the 90th percentile. Observed frequency at each completed week (blue dots). Red lines plotted at 10%, the “ideal” expected frequency. IG‐21st, INTERGROWTH‐21st Project; NICHD‐U, National Institutes of Child Health and Human Development Fetal Growth Studies Unified Standard; WHO, World Health Organization.

The frequency of exams in the lower tail (EFW <10th percentile) and upper tail (EFW >90th percentile) is plotted across GA in Figures 4 and 5, respectively. With a perfect fit of observations to the norms, these frequencies should be 10% at every GA, represented by the red line on each panel. This expectation was most closely met by the Hadlock standard based on formulas [10]. The other FW standards had somewhat fewer than 10% of exams in the lower tail and more than 10% in the upper tails. All the BW references had almost no exams in the lower tail at early GAs and the three oldest BW references [6, 7, 13] had almost no exams in the upper tail at early GAs.

The entire study sample comprised patients with risk factors for SGA (7297 exams, 35.3%), risk factors for LGA (3719 exams, 18.0%), risk factors for both SGA and LGA (5989 exams, 29.0%), and risk factors for neither (low‐risk subgroup, 3638 exams, 17.6%), as shown in Table 1. We repeated all the above analyses in the low‐risk subgroup as summarized in Table 3. In the low‐risk subgroup, the Hadlock formulas [10] had the best overall fit to our data, manifested by a mean *z*‐score closest to 0, SD of *z* close to 1 (though WHO standard was closer), D‐statistic closest to 0, closest to 10% of exams in the lower tail, and J‐statistics closest to 1.

**TABLE 3:** Comparison of 10 norms for estimated fetal weight in “low‐risk” subgroup (*N* = 3638).

Reference or standard	Year	Source	Type	Mean *z*‐score	Standard deviation of *z*‐score	Kolmogorov–Smirnov D‐statistic	Exams with EFW <10th percentile	Youden J‐statistic at 10th percentile	Exams with EFW >90th percentile	Youden J‐statistic at 90th percentile
Ideal values				0	1	0	2064 (10%)	1	2064 (10.0%)	1
Brenner [6]	1976	Table	BWR	0.27^a^	0.51	0.302	17 (0.5%)^b^, ^d^, ^e^, ^h^, ^i^, ^j^, ^k^, ^l^	0.107	110 (3.0%)^b^, ^d^, ^e^, ^f^, ^h^, ^i^, ^j^, ^k^, ^l^	0.480
Williams [7]	1982	Table	BWR	0.04^a^	0.46	0.226	24 (0.7%)^b^, ^e^, ^f^, ^h^, ^i^, ^j^, ^k^, ^l^	0.151	37 (1.0%)^b^, ^c^, ^f^, ^g^, ^h^, ^i^, ^j^, ^k^, ^l^	0.204
Alexander [13]	1996	Table	BWR	−0.24^a^	0.46	0.289	52 (1.4%)^b^, ^c^, ^d^, ^g^, ^h^, ^i^, ^j^, ^l^	0.314	27 (0.7%)^b^, ^c^, ^f^, ^g^, ^h^, ^i^, ^j^, ^k^, ^l^	0.142
Olsen [24]	2010	Table	BWR	0.37^a^	0.68	0.240	20 (0.5%)^b^, ^d^, ^e^, ^h^, ^i^, ^j^, ^k^, ^l^	0.126	333 (9.1%)^c^, ^d^, ^e^, ^g^, ^h^, ^i^, ^k^	0.911
Duryea [26]	2014	Table	BWR	0.19^a^	0.62	0.207	34 (0.9%)^b^, ^h^, ^i^, ^j^, ^k^, ^l^	0.207	128 (3.5%)^b^, ^d^, ^e^, ^f^, ^h^, ^j^, ^k^, ^l^	0.707
Hadlock [10]	1991	Formulas	FWS	−0.02	0.87	0.074	155 (4.3%)^b^, ^c^, ^d^, ^e^, ^f^, ^g^, ^i^, ^k^, ^l^	0.889	232 (6.4%)^b^, ^c^, ^d^, ^e^, ^f^, ^g^, ^j^, ^k^, ^l^	0.980
Hadlock [10]	1991	Table	FWS	−0.02	0.79	0.092	101 (2.8%)^b^, ^c^, ^d^, ^e^, ^f^, ^g^, ^h^	0.635	176 (4.8%)^b^, ^c^, ^d^, ^e^, ^f^, ^j^, ^k^, ^l^	0.938
WHO [29]	2017	Table	FWS	0.16^a^	0.95	0.091	144 (4.0%)^b^, ^c^, ^d^, ^e^, ^f^, ^g^, ^k^, ^l^	0.807	376 (10.3%)^c^, ^d^, ^e^, ^g^, ^h^, ^i^, ^k^	0.944
INTERGROWTH‐21st [30]	2017	Formulas	FWS	0.60^a^	0.84	0.369	71 (2.0%)^b^, ^c^, ^d^, ^e^, ^f^, ^g^, ^h^, ^i^, ^j^, ^l^	0.295	691 (19.0%)^b^, ^c^, ^d^, ^e^, ^f^, ^g^, ^h^, ^i^, ^j^, ^l^	0.853
NICHD Uniform [32]	2025	Formulas	FWS	0.20^a^	0.82	0.354	93 (2.6%)^b^, ^c^, ^d^, ^e^, ^f^, ^g^, ^h^, ^i^, ^j^, ^l^	0.585	327 (9.0%)^c^, ^d^, ^e^, ^g^, ^h^, ^i^, ^k^	0.958

Abbreviations: BWR, birth weight reference; EFW, estimated fetal weight; FWS, fetal weight standard; NICHD, National Institutes of Child Health and Human Development; WHO, World Health Organization.

^a^ Significantly different than 0, *p* < 0.005, *t*‐test.

^b^ Significantly different than 10%, *p* < 0.005, one‐sample *z*‐test.

^c^ Significantly different than Brenner, *p* < 0.0011, two‐sample *z*‐test.

^d^ Significantly different than Willaims, *p* < 0.0011, two‐sample *z*‐test.

^e^ Significantly different than Alexander, *p* < 0.0011, two‐sample *z*‐test.

^f^ Significantly different than Olsen, *p* < 0.0011, two‐sample *z*‐test.

^g^ Significantly different than Duryea, *p* < 0.0011, two‐sample *z*‐test.

^h^ Significantly different than Hadlock formulas, *p* < 0.0011, two‐sample *z*‐test.

^i^ Significantly different than Hadlock table, *p* < 0.0011, two‐sample *z*‐test.

^j^ Significantly different than WHO, *p* < 0.0011, two‐sample *z*‐test.

^k^ Significantly different than INTERGROWTH‐21st, *p* < 0.0011, two‐sample *z*‐test.

^l^ Significantly different than NICHD Uniform, *p* < 0.0011, two‐sample *z*‐test.

## 4 DISCUSSION

The Hadlock formulas for mean and SD of FW [10] yielded the best overall fit to our EFW distribution: mean *z*‐score 0.08, SD of *z* 1.04, D‐statistic 0.036, 7.2% and 11.6% of exams in the lower and upper tails, respectively, and J‐statistics 0.94 and 0.97 at the 10th and 90th percentiles, respectively. The Hadlock table [10] had a similar mean but larger range of normal, resulting in a smaller percentage of exams in both tails.

All the BW references had poor fit to our EFW distribution compared to all the FW standards even though their mean FWs were generally similar to the means of the FW standards; the poor fit was mainly due to the much larger range of normal of the BW standards whether the range is expressed as the absolute difference between the 10th and 90th percentiles or as a percentage of mean FW (Figure 2, lower left and lower right panels, respectively). With a wide range of normal, relatively few observations will fall outside the normal range, as seen in Figures 4 and 5, where the BW references yielded almost no abnormal values at early GA. These findings corroborate the SMFM recommendation that EFW percentiles should be based on FW standards, not on BW standards [34].

It is somewhat surprising that our EFW distribution more closely matched the Hadlock standard [10] than the NICHD‐U standard [32]. Although both standards were derived from US populations, the Hadlock standard was derived from “392 predominantly middle‐class white women” [10] examined at a single site in the 1980s whereas the NICHD‐U standard was from 1737 women of multiple races examined at 12 sites from 2009 to 2013 [27, 31, 32], which more closely matches our sample. Interestingly, our EFW distribution more closely matched the multinational WHO standard [29] than either the multinational IG‐21st [30] or the NICHD‐U standard [32]. The worse fit of the WHO standard [29] compared to the Hadlock standard [10] was mainly due to an excess of estimates >90th percentile when using the WHO standard (Tables 2 and 3, upper tail of WHO histogram in Figure 2); we speculate that this may have been due to the relatively high rate of obesity in our population (Table 1).

It is possible that it is merely a coincidence that a 1985 formula from Hadlock et al. [3] was used to calculate EFW and 1991 formulas from the same group [10] produced the best fit to our EFW distribution. The 1985 and 1991 formulas were derived from different, though overlapping, patient populations. The 1985 formula yields an EFW in grams calculated from fetal measurements (HC, AC, FL) while the 1991 formulas yield an EFW percentile based only on GA, independent of fetal biometry. The 1991 Hadlock FW standard [10] can be used with EFW calculated by any method and does not specify that EFW must be calculated from Hadlock formulas. We used the 1985 Hadlock three‐parameter formula [3] for EFW because other investigations reported that it provided the most accurate prediction of BW [2, 4]. Our study does not evaluate whether a given EFW is accurate, but rather evaluates how well the observed distribution of EFWs fits the expected distribution.

When using commercial ultrasound reporting software to calculate EFW percentiles, it is important for users to know whether the calculation is based on formulas or tables, especially for the Hadlock FW standard [10] for which the percentile cut‐offs in the published table are different than those calculated from the formulas [32, 40]. In Viewpoint software, for example, EFW percentiles for the Hadlock FW standard [10] are based on tables, not formulas. Our findings confirm prior observations that the tables result in a relative underdiagnosis of FGR (EFW <10th percentile) compared to the formulas [32, 40]. We encourage software developers to use the formulas instead, or to make available the revised tables of Roberts et al. [40] which correspond more closely to the Hadlock formulas [10]. For the NICHD‐U standard [31], Grantz et al. recently reported that the tables and formulas agree closely [32]; we similarly found that the formulas yield similar means and percentile cut‐offs to those in the tables (data not shown). We also found that the IG‐21st formulas yield similar values to those in the IG‐21st tables (data not shown).

Strengths of the study include a relatively large sample of contemporary exams from several MFM practices, enhancing generalizability of the findings to other USA MFM practices. A strength of the *z*‐score method is that it provides an objective assessment that can readily be used to evaluate any new or existing norms for EFW or fetal measurements.

A limitation is that we studied a referred population enriched for abnormal fetal growth, both LGA and SGA. Although we present the low‐risk subgroup as an approximation of a standard population, we recognize that it is not a true standard population. Standard norms are developed in studies where low‐risk patients have scheduled exams at prescribed GA time points whereas our study involves a cross‐section of exams at whatever GA the patients were referred for evaluation. Another limitation is the high percentage of exams with missing information about race; although we attempt to collect this, a high percentage of our patients decline to report.

Finally, we have no data on perinatal outcome, so we are unable to correlate EFW percentiles with BW percentiles or neonatal morbidity. Many MFM practices will have a similar limitation; a concerted effort is required to obtain BW, delivery date, and neonatal data on the exams we perform, and even with effort, outcomes cannot be obtained in a substantial fraction of exams [5]. A series of studies by Blue et al. [5, 42, 43, 44, 45] have reported perinatal outcomes associated with the Hadlock FW standard [10] compared to various other norms: In one study [42], the Hadlock standard [10] was superior to NICHD race‐specific FW standards [27] for the prediction of both neonatal morbidity and SGA; in another study [43], the Hadlock standard [10] was superior to the IG‐21st [30] and Salomon et al. [22] FW standards in the prediction of BW percentile; in another study [44], the Hadlock standard [10] was superior to the Olsen BW reference [24] in prediction of neonatal morbidity and SGA, though both norms had poor overall performance to predict morbidity and mortality, especially at term; and another study [45] compared the Hadlock FW standard [10] to a customized standard and found that FGR was diagnosed in 5.5% versus 3.5% of cases, respectively, though both standards had poor performance for prediction of perinatal morbidity. Taking these results together with our findings, we conclude that the Hadlock FW standard [10] is the best of currently known norms for making the diagnosis of FGR.

## 5 CONCLUSIONS

Of the norms studied, the FW distribution from the Hadlock 1991 formulas [10] had the best fit to our observed EFW distribution and is the most appropriate standard for determination of EFW percentiles in our US‐based MFM practices. The Hadlock 1991 tables [10] or their 2025 revision by Roberts et al. [40] may be appropriate surrogates if the Hadlock formulas are not implemented in the reporting software. BW references have a poor fit to the observed EFW distribution because they have a range of normal that is too wide, resulting in underdiagnosis of both FGR and EFW >90th percentile. Other practices, especially those outside the United States, should consider replicating our methods to test these conclusions using their own data.

## AUTHOR CONTRIBUTIONS

**C. Andrew Combs**: Conceptualization; investigation; methodology; data curation; formal analysis; visualization; project administration; writing—original draft. **Olaide Ashimi Balogun**: Writing—review and editing; conceptualization; supervision; validation. **Sushma Amara**: Conceptualization; supervision; writing—review and editing; validation. **Zachary S. Bowman**: Conceptualization; validation; supervision; writing—review and editing. **Peter A. Combs**: Conceptualization; methodology; software; formal analysis; writing—review and editing.

## CONFLICT OF INTEREST STATEMENT

C.A.C. is participating in research with Sonio, Inc. on development of an artificial intelligence algorithm to improve estimation of fetal weight. The other authors declare no conflicts of interest.

## FUNDING INFORMATION

The authors received no specific funding for this work.

## Data Availability

The data that support the findings of this study are available on request from the corresponding author. The data are not publicly available due to privacy or ethical restrictions.
